# Supplementary material for: Noise amplification and ill-convergence of Richardson-Lucy deconvolution
Source: Nat Commun. 2025 Jan 21;16:911. doi: 10.1038/s41467-025-56241-x (PMC11751374; doi:10.1038/s41467-025-56241-x)
Supplement: Supplementary file 1 — Supplementary Information [file 41467_2025_56241_MOESM1_ESM.pdf]

Supplementary Information to "Noise amplification and ill-convergence of Richardson-Lucy deconvolution"

Yiming Liu, Spozmai Panezai, Yutong Wang, and Sjoerd Stallinga

Department of Imaging Physics, Delft University of Technology, Delft, The Netherlands

Theory of Cramér-Rao Lower Bound of Richardson-Lucy deconvolution

Image formation model

We use a framework in which the image formation is modelled such that the object and the images are represented with discrete, finite sized vectors labelled by the discrete pixels, rather than as continuous functions of the coordinates in the object and image planes. As a consequence, the operators that related the object and the image and that represent the image processing steps, are represented as matrices operating in the finite sized vector space that represents the object and image. So, if there are $j = 1,2,\cdots,K$ pixels, the image is represented as the vector $n = (n_1, \dots, n_K)$ and the underlying object as the vector $x = (x_1, \dots, x_K)$. The pixels have a position $\vec{r}_j$ in the object/image plane. For the sake of simplicity we only consider the case of unit magnification between object and image space. The true magnification value can be taken into account by a rescaling of object or image space coordinates. We will frequently make use of the Fourier Transform (FT), transforming a real space vector x into a spatial frequency space vector $\hat{x} = Ux$, and the inverse FT, transforming a spatial frequency space vector $\hat{x}$ into a real space vector $x = U^{-1}\hat{x}$, where the Fourier matrix U and inverse Fourier matrix $U^{-1} = U^{\dagger}/K$ are defined by:

$$U_{jk} = e^{-2\pi i\vec{q}_j\cdot\vec{r}_k} \tag{1}$$

$$U_{kj}^{-1} = \frac{1}{K}e^{2\pi i\vec{q}_j\cdot\vec{r}_k} \tag{2}$$

where the $\vec{q}_j$ represent the positions in spatial frequency space of the pixels in the Fourier domain.

Consider now an incoherent optical imaging system. The expected photon count in the image plane is then the vector $\mu = (\mu_1, \dots, \mu_K) = gx$, where the matrix g represents the Point Spread Function (PSF). We assume that the PSF is positive, $g_{kj} \geq 0$ for all matrix elements, it is normalized to unity, $\sum_{k=1}^{K} g_{kj} = 1$, and we also assume that the imaging is shift invariant, i.e. the PSF only depends on the coordinate differences between the object and image pixels. An important implication is then that the PSF can be expressed as a Fourier series according to:

$$g_{kj} = \frac{1}{K}\sum_{s=1}^{K}\hat{g}_s e^{2\pi i\vec{q}_s\cdot(\vec{r}_k-\vec{r}_j)} \tag{3}$$

where the elements $\hat{g}_s$ represent the Optical Transfer Function (OTF) of the imaging system, and where the shift invariance is manifest. An incoherent optical imaging system is band-limited, i.e. it holds that $\hat{g}_s = 0$ for $|\vec{q}_s| \geq 2NA/\lambda$, with NA the numerical aperture and λ the imaging wavelength. The PSF can be expressed in compact operator form as:

$$g = U^{-1}\hat{g}U \tag{4}$$

where $\hat{g} = \mathrm{diag}(\hat{g}_k)$ is a diagonal matrix. The Fourier transforms $\hat{\mu} = U\mu$ and $\hat{x} = Ux$ are related by $\hat{\mu} = \hat{g}\hat{x}$, which boils down to an element wise multiplication of the object spatial frequency component $\hat{x}_k$ with the OTF component $\hat{g}_k$ to get the expected image spatial frequency component $\hat{\mu}_k$.

Richardson-Lucy deconvolution algorithm

The task is to find the object vector x which best fits the actually observed measured photon counts n. This best fit is defined as the one that optimizes the likelihood that takes into account the noise

statistics. The suitable log-likelihood that interpolates between the pure shot noise and pure readout noise conditions is[1]:

$$\log L(n|x) = \sum_{k=1}^{K} \left[(n_k + \sigma^2) \log(\mu_k + \sigma^2) - (\mu_k + \sigma^2) - \log\big(\Gamma(n_k + \sigma^2 - 1)\big) \right] \tag{5}$$

with σ^2 the readout noise variance and $\Gamma(x)$ the Gamma-function. In the limit $\sigma \downarrow 0$ this form reduces to the shot noise only log-likelihood, whereas in the limit $\sigma \gg 1$ it reduces to the least-squares form that corresponds to Gaussian readout noise. The reasons for considering this mixed Poisson-Gaussian log-likelihood is that modern sCMOS cameras have a small but non-zero contribution from readout noise to the total noise, and that the additional Gaussian noise component can relatively easily be incorporated into the Richardson-Lucy algorithm. The expectation value of a function of n over the statistical distribution is defined as:

$$\langle f \rangle = \sum_{n} L(n|x) f(n) \tag{6}$$

Using the mixed Poisson-Gaussian distribution we find in particular that

$$\langle n_k \rangle = \mu_k \tag{7}$$

$$\langle n_k n_l \rangle = \mu_k \mu_l + (\mu_k + \sigma^2)\delta_{kl} \tag{8}$$

The first order derivatives of the log-likelihood w.r.t. the to-be-optimized parameters x_j are:

$$\frac{\partial \log L(n|x)}{\partial x_j} = \sum_{k=1}^{K} \frac{n_k - \mu_k}{\mu_k + \sigma^2} g_{kj} = -1 + \sum_{k=1}^{K} \frac{n_k + \sigma^2}{\mu_k + \sigma^2} g_{kj} \tag{9}$$

where we have used the normalization of the PSF to unity. A local iterative update algorithm that optimizes the log-likelihood (provided it converges) is:

$$x_j^{(l+1)} = x_j^{(l)} + \beta_j^{(l)} \left(\frac{\partial \log L}{\partial x_j} \right)_{x_j^{(l)}} \tag{10}$$

with $\beta_j^{(l)} > 0$ a coefficient that can be chosen, in principle, arbitrarily, and where $l = 1,2,\cdots$ labels the estimate just prior to iteration l. Hence, $l = 1$ indicates the initial estimate, $l = 2$ indicates the result of the first update, just prior to the second iteration, etc. Up to first order the log-likelihood increases as:

$$\delta \log L = \sum_{j=1}^{K} \left(\frac{\partial \log L}{\partial x_j} \right)_{x_j^{(l)}} \left(x_j^{(l+1)} - x_j^{(l)} \right) = \sum_{j=1}^{K} \beta_j^{(l)} \left(\left(\frac{\partial \log L}{\partial x_j} \right)_{x_j^{(l)}} \right)^2 > 0 \tag{11}$$

According to Richardson-Lucy (RL) we should choose $\beta_j^{(l)} = x_j^{(l)}$ which gives as update rule:

$$x_j^{(l+1)} = \sum_{k=1}^{K} \frac{n_k + \sigma^2}{\mu_k + \sigma^2} g_{kj} x_j^{(l)} \tag{12}$$

where it is implicitly assumed that the values $x_j^{(l)}$ are used in the forward model calculation of the μ_k. The major advantage of the algorithm is positivity by design. If all $x_j^{(l)} \geq 0$ then so will all $x_j^{(l+1)} \geq 0$, as all quantities involved in the update are positive too. In the limiting case $\sigma \downarrow 0$ we retrieve the standard RL update rule.

Lower bound variance and Fisher matrix

Consider an estimator $\hat{\phi}(n)$ of a complex parameter $\phi = \phi(x')$ with $x' = \hat{P}\hat{x} = \hat{P}Ux$. Here $\hat{P}$ is the projection operator on the support of the OTF in Fourier space:

$$\hat{P}_{jk} = \begin{cases} \delta_{jk}, & |\vec{q}_j| = |\vec{q}_k| < 2NA/\lambda \\ 0, & \text{otherwise} \end{cases} \tag{13}$$

This means that the estimated parameter depends only on the Fourier-components $\hat{x}_k$ within the OTF support, that is for $|\vec{q}_k| < 2NA/\lambda$. Furthermore, we assume that the estimator is unbiased, i.e. $\langle\hat{\phi}(n)\rangle = \phi(x')$.

The derivation of the multi-parameter CRLB is usually based on considering the expectation value of the quadratic form:

$$V = \langle \left| \hat{\phi}(n) - \phi(x') - \sum_{j=1}^{K} c_j \frac{\partial \log L(n|x)}{\partial x_j} \right|^2 \rangle \tag{14}$$

that satisfies $V \geq 0$ for any set of complex coefficients c_j. This may be written in compact operator form as:

$$V = \langle|\Delta\phi|^2\rangle - \left(ce^\dagger + ec^\dagger\right) + cFc^\dagger \geq 0 \tag{15}$$

with the variance:

$$\langle|\Delta\phi|^2\rangle = \langle\left|\hat{\phi}(n) - \phi(x')\right|^2\rangle \tag{16}$$

the Fisher-matrix F with components:

$$F_{jk} = \langle \frac{\partial \log L(n|x)}{\partial x_j} \frac{\partial \log L(n|x)}{\partial x_k} \rangle \tag{17}$$

the row vector c with components c_j, and the row vector e with components:

$$\begin{aligned} e_j &= \langle \left(\hat{\phi}(n) - \phi(x')\right) \frac{\partial \log L(n|x)}{\partial x_j} \rangle = \sum_n \left(\hat{\phi}(n) - \phi(x')\right) \frac{\partial L(n|x)}{\partial x_j} \\ &= \frac{\partial}{\partial x_j}\left[\sum_n \left(\hat{\phi}(n) - \phi(x')\right) L(n|x)\right] - \sum_n \frac{\partial\left(\hat{\phi}(n) - \phi(x')\right)}{\partial x_j} L(n|x) \\ &= \frac{\partial}{\partial x_j}\left[\langle\hat{\phi}(n)\rangle - \phi(x')\right] + \frac{\partial \phi(x')}{\partial x_j} = \frac{\partial \phi(x')}{\partial x_j} \end{aligned} \tag{18}$$

where the normalization condition on the likelihood and the unbiased nature of the estimator are used. The row vector e can be expressed in operator form as:

$$e = \frac{\partial \phi(x')}{\partial x} = \frac{\partial \phi(x')}{\partial x'} \hat{P} U \equiv e' \hat{P} U \tag{19}$$

giving the column vector $e^\dagger = U^\dagger \hat{P} e'^\dagger$. An explicit expression for the Fisher matrix can be derived from Eqs. (7), (8), (9), and (17) as:

$$F_{ij} = \sum_{k=1}^{K} \frac{g_{ki} g_{kj}}{\mu_k + \sigma^2} \tag{20}$$

This may be expressed in operator notation as:

$$F = g^T M^{-1} g = g^\dagger M^{-1} g \tag{21}$$

where M is a diagonal matrix:

$$M = \mathrm{diag}(\mu_k + \sigma^2) \tag{22}$$

and where we used that $g = g^*$ so that $g^T = g^\dagger = U^{-1} \hat{g}^* U$.

Regularization procedure for computing the CRLB

The expected next step might be to look for the strictest lower bound for V, which would yield an expression for the CRLB in terms of the Moore-Penrose pseudo-inverse F^+ of the Fisher matrix:

$$\langle|\Delta\phi|^2\rangle \geq eF^+e^\dagger \tag{23}$$

It appears, however, that the pseudo-inverse of an operator of the form of Eq. (20) cannot be easily found. Instead, we will follow a route of regularization, which avoids the computation of the pseudo-inverse, but still enables the computation of the CRLB, at least for the non-null space of Fourier components within the OTF support. The regularized Fisher matrix is defined as:

$$\tilde{F} = U^{-1}\left(\hat{g}^*\hat{P} + \epsilon\left(I - \hat{P}\right)\right)UM^{-1}U^{-1}\left(\hat{g}\hat{P} + \epsilon\left(I - \hat{P}\right)\right)U \tag{24}$$

for $0 < \epsilon \ll 1$, which is equal to the true Fisher-matrix in the limit $\epsilon \downarrow 0$. We will in the end take this limit in the final expression for the CRLB of the $\hat{x}_k$ for $|\vec{q}_k| < 2NA/\lambda$. The regularized quantity:

$$\tilde{V} = \langle|\Delta\phi|^2\rangle - \left(ce^\dagger + ec^\dagger\right) + c\tilde{F}c^\dagger \tag{25}$$

satisfies $\lim_{\epsilon\to 0} \tilde{V} \geq 0$. The minimum of $\tilde{V}$ is found when the coefficients c satisfy $c\tilde{F} = e$, implying that $c = e\tilde{F}^{-1}$. Then it holds that:

$$\tilde{V} = \langle|\Delta\phi|^2\rangle - e\tilde{F}^{-1}e^\dagger \tag{26}$$

and the lower bound for the variance of the unbiased estimator is:

$$\langle|\Delta\phi|^2\rangle \geq \lim_{\epsilon\to 0} e\tilde{F}^{-1}e^\dagger = \lim_{\epsilon\to 0} e'\hat{P}U\tilde{F}^{-1}U^\dagger\hat{P}e'^\dagger \tag{27}$$

The regularized Fisher matrix has an inverse:

$$\tilde{F}^{-1} = U^{-1}\left(\hat{g}^{-1}\hat{P} + \frac{1}{\epsilon}\left(I - \hat{P}\right)\right)UMU^{-1}\left(\hat{g}^{*-1}\hat{P} + \frac{1}{\epsilon}\left(I - \hat{P}\right)\right)U \tag{28}$$

This leads to:

$$\hat{P}U\tilde{F}^{-1}U^\dagger\hat{P} = K\hat{g}^{-1}\hat{P}U^{-1}MU\hat{P}\hat{g}^{*-1} \tag{29}$$

where we use that $U^\dagger = KU^{-1}$, and results in a lower bound:

$$\langle|\Delta\phi|^2\rangle \geq \lim_{\epsilon\to 0} Ke'\hat{g}^{-1}\hat{P}U^{-1}MU\hat{P}\hat{g}^{*-1}e'^\dagger = Ke'\hat{g}^{-1}\hat{P}U^{-1}MU\hat{P}\hat{g}^{*-1}e'^\dagger \tag{30}$$

where the limit $\epsilon \downarrow 0$ can be left out as no terms that depend on the small positive parameter ϵ remain. Using that:

$$[U^{-1}MU]_{kl} = \frac{\hat{\mu}(\vec{q}_k - \vec{q}_l)}{K} + \sigma^2\delta_{kl} \tag{31}$$

we find:

$$\langle|\Delta\phi|^2\rangle \geq \sum_{|\vec{q}_k|,|\vec{q}_l|<2NA/\lambda} \frac{\hat{\mu}(\vec{q}_k - \vec{q}_l) + K\sigma^2\delta_{kl}}{\hat{g}_k\hat{g}_l^{\,*}}\left(\frac{\partial\phi}{\partial\hat{x}_k}\right)\left(\frac{\partial\phi}{\partial\hat{x}_l}\right)^* \tag{32}$$

Suppose now we would have looked at the estimation of $\phi = \phi(x')$ with $x' = \left(I - \hat{P}\right)\hat{x}$, i.e. the estimation of a parameter that depends on object spatial frequency components outside the OTF support. Then the $1/\epsilon$ terms would remain, leading to a non-existent CRLB in the limit $\epsilon \downarrow 0$. This signifies the ill-posedness of the problem, as parameters that depend on the Fourier components outside the OTF support can in principle not be inferred from the measured image. For parameter estimation within the OTF support a working solution can be found for the CRLB using the above regularization procedure.

In particular it now follows that the variance of the Fourier transform of the estimate within the OTF support is bounded from below by:

$$\langle|\Delta\hat{x}_k|^2\rangle \geq \left[K\hat{g}^{-1}\hat{P}U^{-1}MU\hat{P}\hat{g}^{*-1}\right]_{kk} = K\frac{\mu_{av}+\sigma^2}{|\hat{g}_k|^2} \tag{33}$$

where we define $\mu_{av} = \hat{\mu}(0)/K$, the average expected photons per pixel. The CRLB Eq. (33) can also be formulated in terms of the Spectral Signal-to-Noise Ratio (SSNR) of the deconvolved image:

$$SSNR_k = \frac{|\hat{x}_k|^2}{\langle|\Delta\hat{x}_k|^2\rangle} \leq \frac{|\hat{g}_k|^2|\hat{x}_k|^2}{K(\mu_{av}+\sigma^2)} = \frac{|\hat{\mu}_k|^2}{K(\mu_{av}+\sigma^2)} \tag{34}$$

The total SNR summed over Fourier space is limited by:

$$SNR \equiv \sum_k SSNR_k \leq \frac{\sum_k|\hat{\mu}_k|^2}{K(\mu_{av}+\sigma^2)} \tag{35}$$

The implication is that, provided the MLE procedure converges to an optimum, the SSNR cannot be higher than it was for the original raw image acquisition.

We can draw even farther reaching conclusions concerning noise amplification in RL-deconvolution. This is expected when the OTF is small, and in the limiting case when the OTF cutoff is approached:

$$\lim_{|\vec{q}_k|\to 2NA/\lambda}\langle|\Delta\hat{x}_k|^2\rangle \geq \lim_{|\vec{q}_k|\to 2NA/\lambda} K\frac{\mu_{av}+\sigma^2}{|\hat{g}_k|^2} = \infty \tag{36}$$

Using Parseval's theorem we find in addition for the noise variance in real space averaged across the image:

$$\frac{1}{K}\sum_{j=1}^{K}\langle|\Delta x_j|^2\rangle = \frac{1}{K^2}\sum_{k=1}^{K}\langle|\Delta\hat{x}_k|^2\rangle \geq \frac{1}{K^2}\sum_{|\vec{q}_k|<2NA/\lambda}\langle|\Delta\hat{x}_k|^2\rangle \tag{37}$$

Combining with the CRLB gives:

$$\frac{1}{K}\sum_{j=1}^{K}\langle|\Delta x_j|^2\rangle \geq (\mu_{av}+\sigma^2)\left(\frac{1}{K}\sum_{|\vec{q}_k|<2NA/\lambda}\frac{1}{|\hat{g}_k|^2}\right) \tag{38}$$

The average over the inverse squared OTF (the term between round brackets in the lower bound) is much larger than one showing that the lower bound on the average noise variance across all image pixels is very large indeed, in any case much larger than the average detected photon count across all pixels μ_{av}. Note that the contribution from the out-of-band noise is not even taken into account in this inequality. In the limit of a large Field Of View (FOV) the number of pixels is very large and in this limit we find:

$$\lim_{K\to\infty}\frac{1}{K}\sum_{j=1}^{K}\langle|\Delta x_j|^2\rangle \geq (\mu_{av}+\sigma^2)\lim_{K\to\infty}\frac{1}{K}\sum_{|\vec{q}_k|<2NA/\lambda}\frac{1}{|\hat{g}_k|^2}$$
$$= (\mu_{av}+\sigma^2)\int_0^{2NA/\lambda} q\,dq\int_0^{2\pi} d\psi\frac{1}{|\hat{g}(\vec{q})|^2} = \infty \tag{39}$$

The integral diverges because $\hat{g}(\vec{q})\sim(2NA/\lambda-|\vec{q}|)^{3/2}$, close to the cutoff.

Additional references

1. Huang, F. et al. Video-rate nanoscopy using sCMOS camera–specific single-molecule localization algorithms. *Nature Methods* **10**, 653-658 (2013).

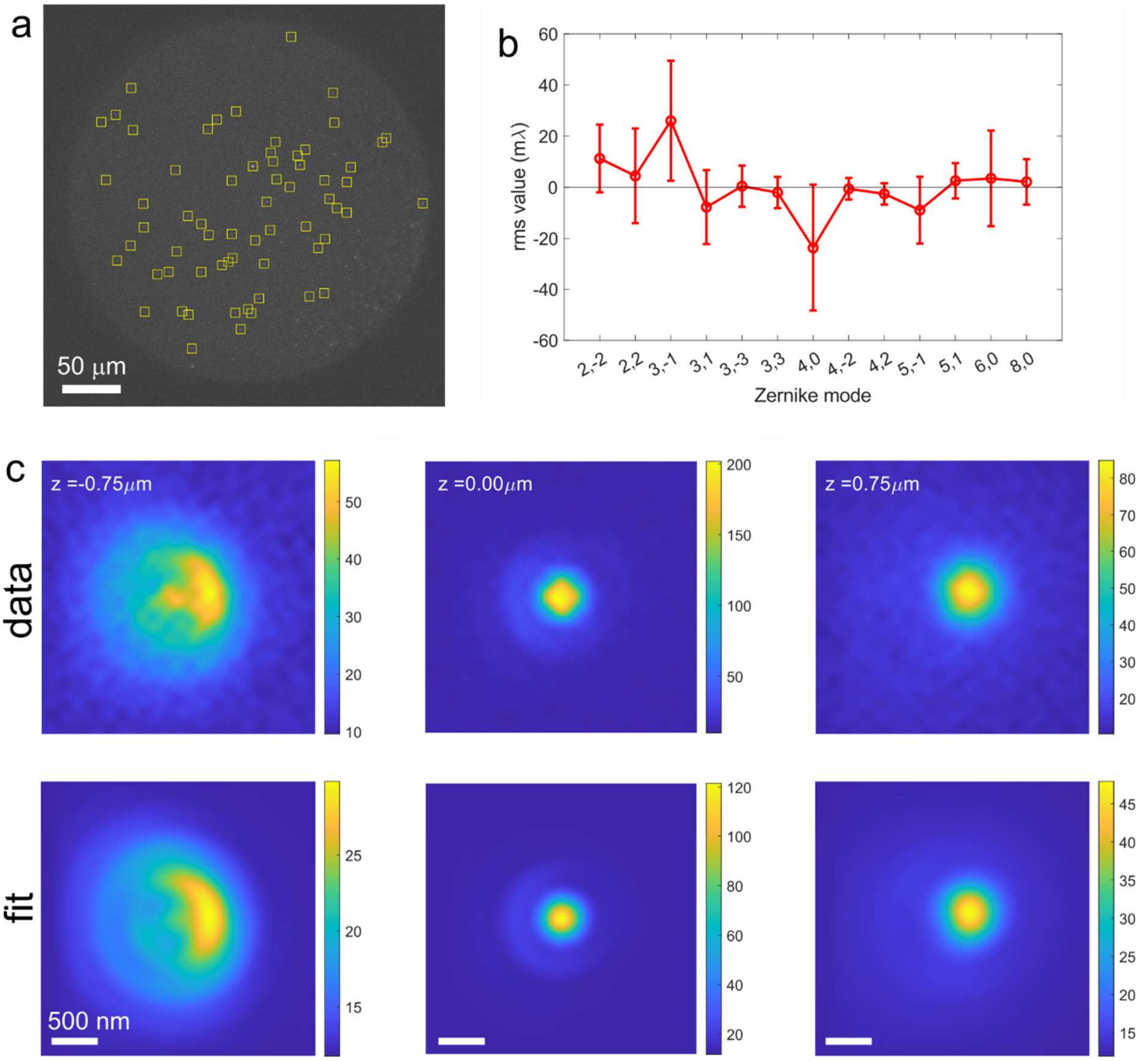

Supplementary Figure 1 | Results from PSF analysis. (a) Selected beads across the Field Of View for a through-focus based aberration estimation. (b) Resulting set of Zernike coefficients, indicating coma and spherical aberration as dominant aberrations. (c) Three instances of measured and fitted PSF, averaged over all 65 selected beads, confirming the presence of coma and spherical aberration (full through-focus data in Movie 3). Scale bar (a) 50 µm, Scale bar (c) 500 nm.

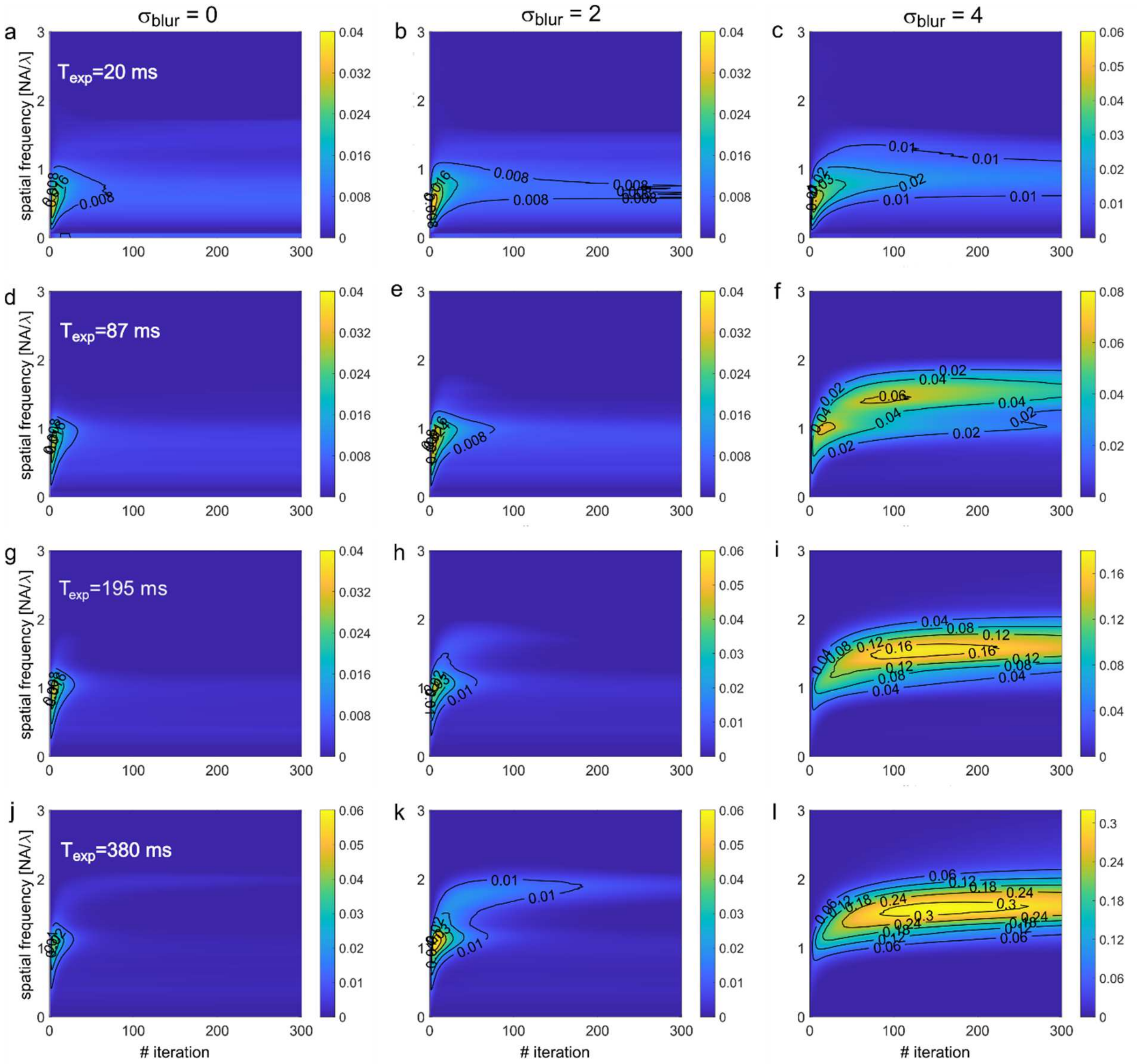

Supplementary Figure 2 | Increase in SSNR during RL deconvolution, for different exposure times (rows) and PSF blurring factors (columns) for the mitochondria channel. A non-linear mapping $\log_{10}(1 + SSNR)$ is used to better visualize small differences.

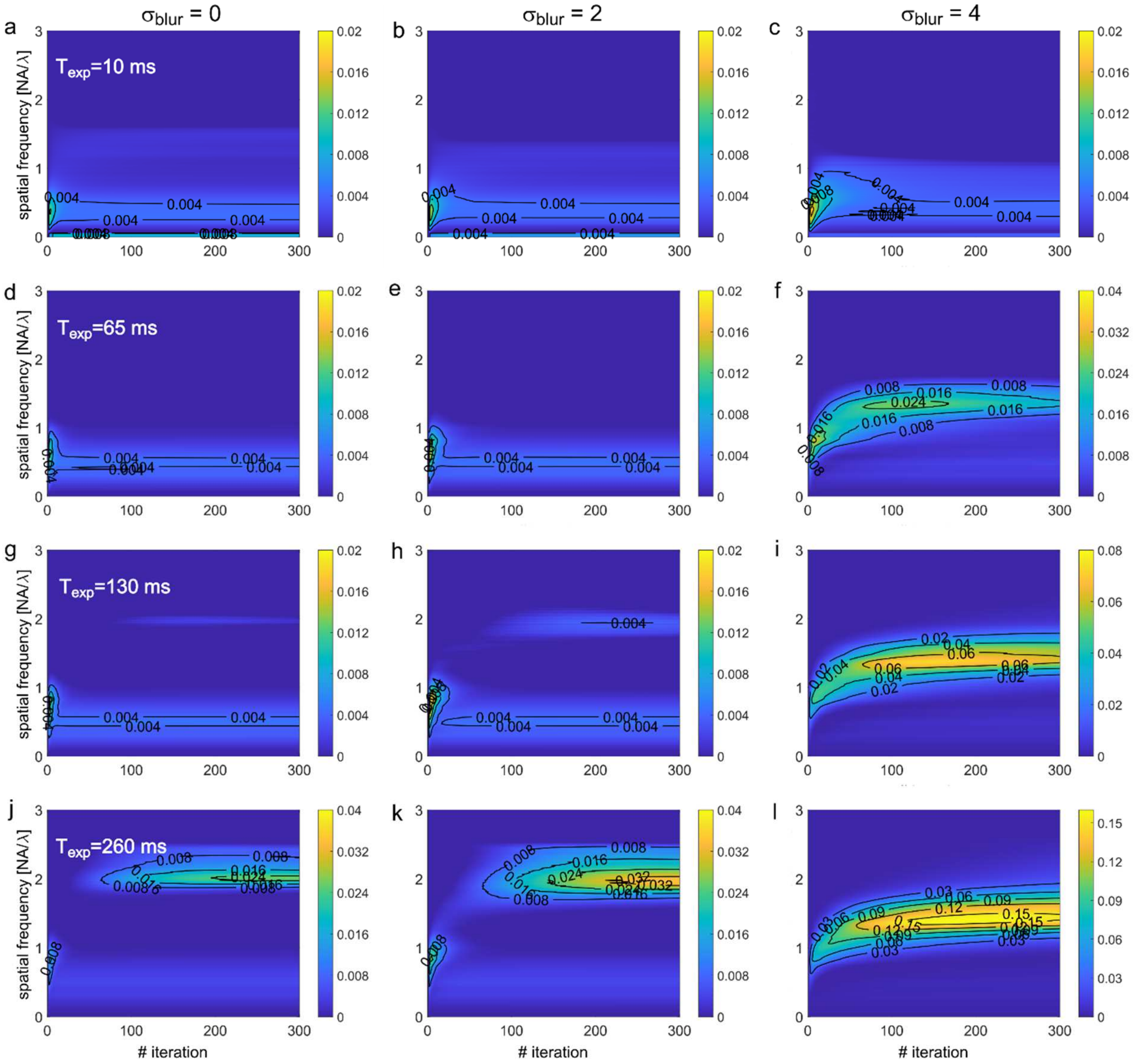

Supplementary Figure 3 | Increase in SSNR during RL deconvolution, for different exposure times (rows) and PSF blurring factors (columns) for the actin channel. A non-linear mapping $\log_{10}(1 + SSNR)$ is used to better visualize small differences.
