# Supplementary material for: Noise amplification and ill-convergence of Richardson-Lucy deconvolution
Source: Nat Commun. 2025 Jan 21;16:911. doi: 10.1038/s41467-025-56241-x (PMC11751374; doi:10.1038/s41467-025-56241-x)
Supplement: Supplementary file 2 — Description of Additional Supplementary Files [file 41467_2025_56241_MOESM2_ESM.pdf]

Description of Additional Supplementary Files

Supplementary Movie 1: Mean and spread of deconvolution in real and Fourier space of mitochondria dataset as a function of iteration number.

Supplementary Movie 2: Mean and spread of deconvolution in real and Fourier space of actin dataset as a function of iteration number.

Supplementary Movie 3: Through-focus data and fit of bead calibration for OTF determination.

Supplementary Movie 4: Simulation of impact of OTF model mismatch on deconvolution noise amplification.
“cameraman” image by Unknown creator, 1978, taken from MIT Libraries – Dome. URL: https://hdl.handle.net/1721.3/195767.

Accessed: January 2025.

The first known appearance of this image was in William F Schreiber's "Image Processing for Quality Improvement" in the Proceedings of IEEE, Vol. 6, No. 12, December 1978.

Licensed under a Creative Commons Attribution Non-Commerical license. For use in journal publications or trade and educational book publishers that might fall outside the CC license terms, the terms provided in the additional download apply (see Cameraman Non-CC TOU on https://hdl.handle.net/1721.3/195767). https://creativecommons.org/licenses/by-nc/4.0/
